# Iron Supplementation during Three Consecutive Days of Endurance Training Augmented Hepcidin Levels

**DOI:** 10.3390/nu9080820

**Published:** 2017-07-30

**Authors:** Aya Ishibashi, Naho Maeda, Akiko Kamei, Kazushige Goto

**Affiliations:** 1 Department of Sports Science, Japan Institute of Sports Science, Nishigaoka, Kitaku, Tokyo 115-0056, Japan; aya.ishibashi@jpnsport.go.jp (A.I.); akiko.kamei@jpnsport.go.jp (A.K.); 2 Graduate School of Sport and Health Science, Ritsumeikan University, Kusatsu, Shiga 525-8577, Japan; maeda.nh0709@gmail.com

**Keywords:** iron related-hormone, endurance training, iron supplementation

## Abstract

Iron supplementation contributes an effort to improving iron status among athletes, but it does not always prevent iron deficiency. In the present study, we explored the effect of three consecutive days of endurance training (twice daily) on the hepcidin-25 (hepcidin) level. The effect of iron supplementation during this period was also determined. Fourteen male endurance athletes were enrolled and randomly assigned to either an iron-treated condition (Fe condition, *n* = 7) or a placebo condition (Control condition; CON, *n* = 7). They engaged in two 75-min sessions of treadmill running at 75% of maximal oxygen uptake on three consecutive days (days 1–3). The Fe condition took 12 mg of iron twice daily (24 mg/day), and the CON condition did not. On day 1, both conditions exhibited significant increases in serum hepcidin and plasma interleukin-6 levels after exercise (*p* < 0.05). In the CON condition, the hepcidin level did not change significantly throughout the training period. However, in the Fe condition, the serum hepcidin level on day 4 was significantly higher than that of the CON condition (*p* < 0.05). In conclusion, the hepcidin level was significantly elevated following three consecutive days of endurance training when moderate doses of iron were taken.

## 1. Introduction

Iron deficiency (ferritin <20 ng/mL) is frequently observed among endurance athletes [1], as it is more common than iron-deficient anemia and affects 13–22% of elite athletes [2,3]. Several physiological mechanisms have been proposed to explain this phenomenon, including gastrointestinal bleeding [4], hemolysis [5], lack of dietary iron [6], and iron loss in sweat [7]. Additionally, the regulatory hormone hepcidin may be involved [8]. Hepcidin, a 25-amino acid peptide, is a crucial mediator of iron homeostasis and may be associated with iron deficiency in response to exercise training. Iron is taken up by enterocytes and is either bound to transferrin or stored as intracellular ferritin [9]. Hepcidin internalizes (degrades) the ferroportin export channels of the small intestine and macrophage surface, inhibiting gut absorption of dietary iron and preventing iron release by macrophages [10]. Hepcidin expression is upregulated by increased iron intake and/or storage [10,11] and inflammation [12,13]. In contrast, it is downregulated by iron deficiency anemia and hypoxia [14].

A low ferritin level with iron supplementation did not affect endurance performance (e.g., running economy, time to exhaustion) in iron-deficient non-anemic athletes [15,16]. In contrast, iron treatments may improve the iron status and endurance performance even in iron-deficient non-anemic athletes [17], although these findings are not consistent. Therefore, the question of whether iron supplementation during intense training might improve endurance performance has not been fully explored [18]. A high dose of iron supplementation can be applied among some competitive athletes [19,20], but high doses of iron supplementation may stimulate hepcidin production to maintain iron homeostasis [10,11]. Such high-level supplementation (60–240 mg daily) increased the serum hepcidin level after 24 h, and the fractional iron absorption fell by 35–45% [21]. However, the influence of moderate iron supplementation during strenuous training on the hepcidin level remains unclear. This is an important issue because iron supplementation is a widespread practice among endurance athletes.

Previous studies exploring the influence of acute exercise on iron metabolism found that the hepcidin level was transiently elevated about 3 h after exercise [8,22,23,24] and that this was associated with increases in exercise-induced inflammation and hemolysis [24]. Strenuous exercise promotes inflammation, as reflected by a marked increase in the interleukin-6 (IL-6) level [25]. Pro-inflammatory cytokines (e.g., IL-6) stimulate hepcidin production [10,26,27], and sustained inflammation caused by cumulative exercise may promote hepcidin production and iron deficiency. Peeling et al. [28] recently reported that resting iron status, in addition to post-exercise IL-6 level and exercise intensity, accounted for ~77% of the variance in post-exercise hepcidin elevation in elite athletes. 

Two recent studies found that seven days of running and/or military training, followed by a 54-km skiing event, significantly increased the basal hepcidin level [29,30]. Furthermore, we previously observed that augmented monthly training significantly increased the hepcidin level in long-distance runner [31]. In contrast, other studies found that exercise did not significantly influence the hepcidin level [23,32]. However, the effects of several consecutive days of strenuous endurance training have not yet been determined.

In the present study, we investigated the impact of three consecutive days of endurance training on the hepcidin level. Training was performed twice daily, because the typical training programs among endurance athletes involve two daily sessions (with several hours of rest between). The influence of iron supplementation during training was also determined. We hypothesized that three consecutive days of endurance training would elevate the hepcidin level and that iron supplementation during training would further augment this level.

## 2. Materials and Methods

### 2.1. Subjects

Fourteen male endurance athletes (long-distance runners and triathletes) participated (means ± standard errors (SE): age: 19–22 years; height: 1.68 ± 0.01 m; weight: 55.9 ± 1.1 kg; maximal oxygen uptake ($\dot{V}$O_2max_): 59.6 ± 0.8 mL/kg/min). All subjects were healthy and trained regularly on ≥4 days a week. The exclusion criteria were smoking and the use of herbs or medications. All subjects were informed about the study protocol, the possible benefits and risks, and they provided written informed consent. The study was approved by the Ethics Committee for Human Experiments of Ritsumeikan University, Japan (BKC-IRB-2015-023).

### 2.2. Experimental Design

This was a single-blinded placebo-controlled study. The 14 subjects were randomly assigned to either an iron-treatment (Fe condition; *n* = 7) or a placebo (CON condition; *n* = 7) condition; $\dot{V}$O_2max_ level were evaluated prior to the experiment. All subjects completed three consecutive days of twice-daily endurance exercises (75 min bouts of treadmill running at 75% of $\dot{V}$O_2max_ in the morning (Ex 1; 8:30–10:45) and afternoon (Ex 2; 13:00–14:15). The Fe condition received 12 mg of iron in a flavored drink (100 mL) before and immediately after Ex 1 (24 mg/day); CON subjects received the drink only. Iron supplementation among athletes often features 24 mg Fe/day [33]. 

Blood samples were collected from the antecubital vein at 08:00 during the training period (days 1–3) and on the next day (day 4).

During the three days of training, all subjects arrived at the laboratory at 08:00 following an overnight fast. Body weight, fatigue score, and muscle soreness were evaluated using a visual analog scale. All subjects completed the six exercise sessions at 75% of the $\dot{V}$O_2max_. However, the running velocity was reduced when a subject could not maintain the prescribed velocity because of accumulated fatigue. Water was given ad libitum throughout all sessions, and standard meals were provided at 11:00, 13:30, and 19:00. 

### 2.3. Measurements

#### 2.3.1. Determination of Running Velocity

About two weeks prior to the training period, an incremental treadmill exercise test (Life Fitness 95T, Chicago, IL, USA) was used to assess $\dot{V}$O_2max_. The initial running velocity was 6 km/h and was increased progressively by 2 km/h every minute. When the velocity attained 14.6 km/h, it was further increased by 0.6 km/h every minute to exhaustion. The treadmill gradient was 0% (flat) [34]. Respiratory gases were collected using a breath-by-breath methods; we evaluated oxygen uptake ($\dot{V}$O_2_), carbon dioxide output ($\dot{V}$CO_2)_, ventilation volume ($\dot{V}$E), and the respiratory exchange ratio (RER) using a metabolic cart (AE300S, Minato Medical Science Co., Osaka, Japan). The oxygen, carbon dioxide, and flow sensors were calibrated before each test according to the manufacturer’s instructions. The exercise test was terminated when the subject could not maintain the prescribed running speed or when the $\dot{V}$O_2_ plateau was attained. The running velocity and $\dot{V}$O_2_ were used to calculate the running speed associated with 75% of the $\dot{V}$O_2max_.

#### 2.3.2. Blood Sampling and Analyses

Resting blood samples from the antecubital vein were collected after an overnight fast (at least 12 h) during the experimental period (days 1–4) and 3 h after Ex 2 on day 1. Serum and plasma samples were stored at −80 °C after centrifugation for 10 min (3000 rpm, 4 °C). Two-milliliter samples were transferred to ethylenediaminetetraacetic acid (EDTA) containing tubes immediately after sampling for determination of hematological parameters; blood hemoglobin (Hb) levels were measured in a clinical laboratory (Falco Holdings Co., Kyoto, Japan). Serum total iron binding capacity (TIBC) and ferritin, iron, transferrin, creatine kinase (CK), high-sensitivity C reactive protein (hsCRP), and myoglobin levels were measured in another clinical laboratory (SRL Co., Tokyo, Japan). Transferrin saturation (TSAT) was calculated as the serum iron level/serum TIBC level × 100. Plasma IL-6 levels were determined using a commercial enzyme-linked immunosorbent assay (ELISA) kit (R and D Systems Inc., Minneapolis, MN, USA). Serum hepcidin levels were measured by cation-exchange chromatography followed by liquid chromatography-tandem mass spectrometry (LC-MS/MS). 

The intra-assay coefficients of variation (CVs) were as follows: 0.6% for Hb, 3.8% for ferritin, 1.4% for iron, 1.6% for the TIBC, 2.3% for CK, 3.2% for hsCRP, 3.4% for myoglobin, and 5.2% for IL-6.

#### 2.3.3. Scores of Fatigue and Muscle Soreness

Subjective fatigue and muscle soreness levels were evaluated using a visual analog scale (VAS). The subjects were instructed to draw lines on 100 mm scales that were marked with “not tired” or “no pain” on the left and with “extremely tired” or “the worst pain ever” on the right [35].

#### 2.3.4. Nutritional Assessment and Standard Meal

All subjects were asked to maintain their usual dietary intake during the month before commencement of training. Regular food and nutrient consumption were calculated using dedicated software (Excel Eiyo-kun FFQg version 4.0; Kenpaku-sha, Tokyo, Japan). The FFQg yields average intake/week of 29 food groups and 10 forms of cookery in conventional units or portion sizes. No subject took an iron supplement. The standard meals were individually adjusted to reflect the usual food and nutrient consumptions.

#### 2.3.5. Statistical Analyses

All data are presented as means ± SE. Changes over time in blood variables were evaluated using two-way analysis of variance (ANOVA) with repeated measures (condition (Fe, CON) × time (days 1–4)). When the ANOVA revealed a significant interaction or a main effect, Tukey’s post-hoc analysis was performed to explore where the difference was located. In addition to *p*-values, we calculated Cohen’s d-values (on independent *t*-test) or the partial η² values (when a two-way ANOVA with repeated measures was performed) to determine effect size (ES). All analyses were performed with the aid of SPSS version 22.0 software (SPSS Inc, Chicago, IL, USA). A *p*-value < 0.05 was considered to reflect statistical significance.

## 3. Results

### 3.1. General Information during Training Sessions

The running distance and the mean heart rate during exercise are shown in Table 1. There was no significant difference between the Fe and CON conditions for any variables. The total running distance over the three days of training (six exercise sessions) was 101.9 ± 2.6 km in the Fe condition and 98.0 ± 3.3 km in the CON condition; this difference did not reach statistical significance (*p* > 0.05). 

### 3.2. Blood Parameters

#### 3.2.1. Iron Parameter

The Hb level on day 1 did not differ significantly between the conditions (Fe: 15.4 ± 0.3 g/dL, CON: 15.4 ± 0.5 g/dL, *p* > 0.05). Table 2 presents the serum ferritin, iron, and TSAT levels. 

#### 3.2.2. Muscle Damage and Inflammatory Parameters

**Figure 1:**
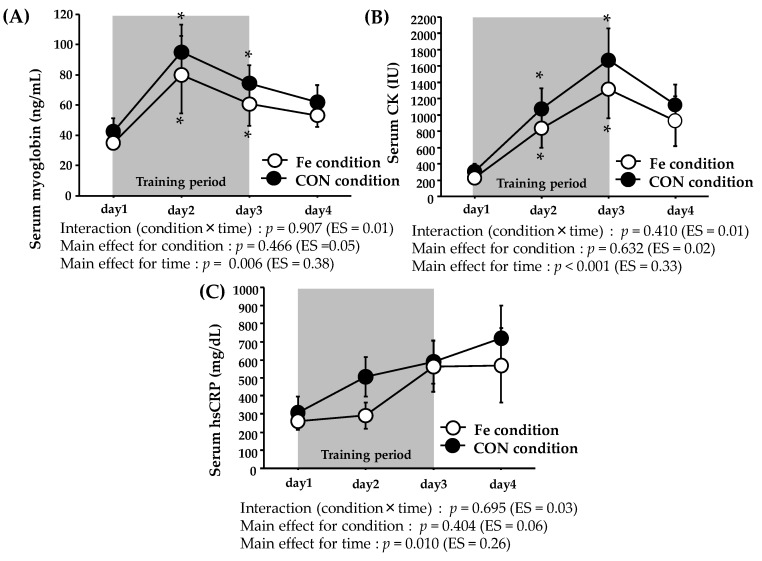
Serum myoglobin (**A**); CK (**B**); and hsCRP (**C**) levels on days 1–4. The values are means ± SE. * Significant difference from day 1.

Figure 1 shows the serum myoglobin, CK and hsCRP levels over time. The myoglobin level exhibited no significant interaction or main effect for condition, and there was a significant main effect for time. The serum CK and hsCRP levels did not show significant interaction or main effect for condition, with a main effect for time. 

**Figure 2:**
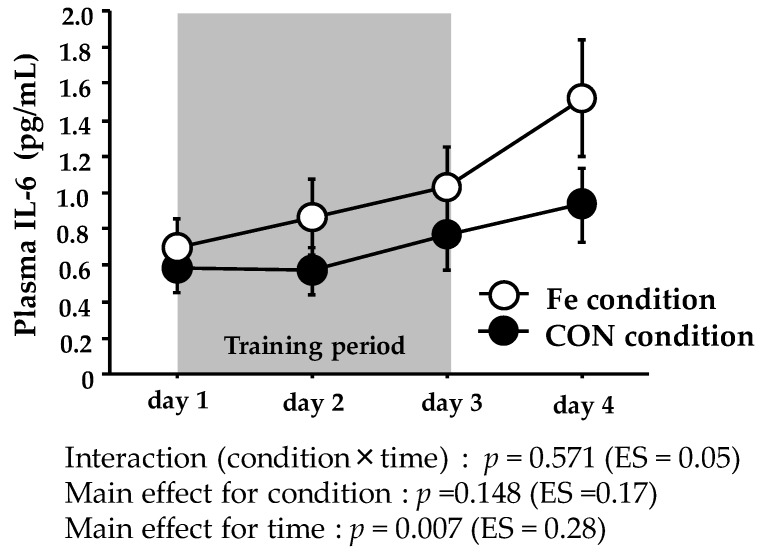
Plasma IL-6 levels during days 1–4. The values are means ± SE.

Figure 2 shows the plasma IL-6 levels. The plasma IL-6 level did not show significant interaction or main effect for condition, with only a significant main effect for time.

#### 3.2.3. Serum Hepcidin Level

**Figure 3:**
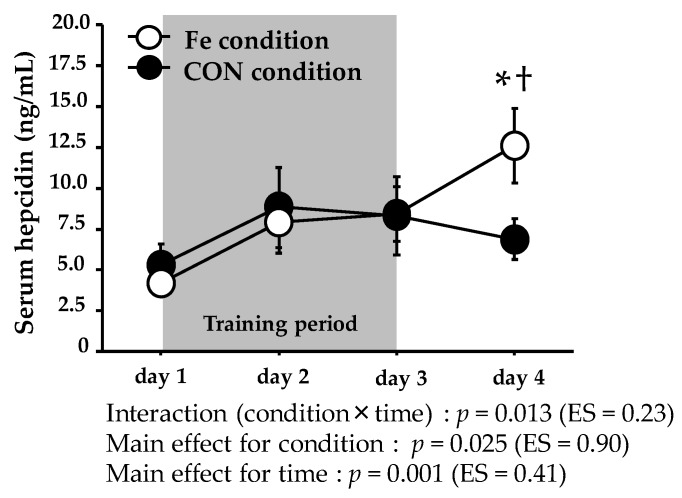
Serum hepcidin levels during days 1–4. The values are means ± SE. * Significant difference from day 1. † Significant difference between conditions.

Figure 3 shows the serum hepcidin levels. The serum hepcidin level exhibited significant interaction (condition × time) over days 1–4. On day 4, the serum hepcidin level was significantly higher in the Fe condition (12.6 ± 1.9 (range: 3.2–20.5) ng/mL) than the CON condition (6.9 ± 1.9 (range: 2.5–14.5) ng/mL).

### 3.3. Score of Fatigue and Muscle Soreness

Table 3 presents the fatigue and muscle soreness scores. The score of fatigue and muscle soreness exhibited no significant interaction or main effect for condition, and there was a significant main effect for time.

### 3.4. Dietary Intake during the Training Periods

Table 4 presents the data on dietary intake. We found no significant differences in energy, carbohydrate, protein, or fat intake during training between the conditions. The average carbohydrate intake over the three days were 7.0 ± 0.2 g/kg (Fe condition) and 6.9 ± 0.3 g/kg (CON condition). Due to iron supplementation during training period, the Fe condition exhibited a significantly higher Fe intake than the CON condition (*p* < 0.001, ES = 2400).

## 4. Discussion

This is the first study to explore the effect of iron supplementation on the hepcidin level during three consecutive days of endurance training. Our principal finding is that three days of training (twice daily) did not significantly change the hepcidin level in the CON condition without iron supplementation. However, in the Fe condition, hepcidin levels were significantly elevated after training accompanied by moderate iron supplementation (24 mg/day). Contrary to our hypothesis, no change in the serum hepcidin level was observed throughout training in the CON condition. Although the hepcidin level is commonly elevated after a single bout of exercise [21,22], the cumulative effect of daily endurance training on the hepcidin level has not previously been fully evaluated. Sim et al. [29] found that that seven consecutive days of running training significantly increased the resting urine hepcidin level. McClung et al. [30] reported that seveb days of winter training (four days of military training followed by three days of cross-country skiing) significantly elevated the serum hepcidin level. Moreover, increases in training intensity and duration elevated the serum hepcidin level in athletes [31,36,37]. In contrast, eight weeks of endurance training (continuous or interval running) did not increase the hepcidin level [32]. Similarly, the serum hepcidin level was not affected by nine weeks of basic combat training in female soldiers [38]. Thus, the cumulative effects of endurance training on the hepcidin level remain unclear. In consequence, our present findings suggest that three consecutive days of such training (75 min of running twice daily) did not strongly impact the serum hepcidin level. Erythropoiesis is one of the stimulating factors of hepcidin expression [39], and hypoxia augments erythropoiesis [40]. However, because all exercises were conducted under normoxic conditions, it is unlikely that erythropoiesis was augmented in the Fe condition.

The exercise-induced increase in the IL-6 level has been suggested to stimulate hepcidin production [41,42]. Although we did not measure IL-6 levels immediately after exercise, several studies have found that the levels become markedly elevated at this time [43,44,45]. The exercise-induced IL-6 elevation was followed by an increase in the hepcidin level, peaking about 3 h later [8,21,22,41,42,45]. On day 1, we observed a significant increase in the hepcidin level 3 h after Ex 2, which was consistent with previous findings (data not shown). We also measured the serum myoglobin and CK levels (indirect markers of muscle damage) because sustained muscle damage and/or inflammation have been suggested to increase the hepcidin level [46]. Both the myoglobin and CK levels were significantly elevated on days 2 and 3 in both conditions and did not differ significantly between the conditions. Furthermore, the myoglobin level peaked on day 2, and the CK level peaked on day 3; it tended to return to normal on day 4. It is, thus, possible that the duration of augmented muscle damage/inflammation was too short to alter the hepcidin level in the CON condition.

Hepcidin production is affected by the energy balance; a lower energy balance and/or depletion of muscle glycogen may increase the hepcidin level, which is attributable to an increase in IL-6 production [47,48]. In a previous study by Badenhorst et al. [49], subjects performed intensive running to deplete muscle glycogen, and they were then given either a low (3 g kg^−1^) or a high (8 g kg^−1^) CHO diet during the next 24 h. On the following day, both the pre- and post-exercise hepcidin levels were significantly elevated in those who had consumed the lower CHO diet. Therefore, high CHO intake may inhibit hepcidin elevation, although any benefit thus afforded was not fully evident in recent studies [49,50,51,52]. In the present study, all subjects consumed prescribed diets during training; the CHO intake was about 7 g kg^−1^/day. However, as training was conducted twice daily (150 min of running per day),the negative CHO balance during training would be equal in the two conditions. Therefore, the elevated hepcidin level on day 4 in the Fe condition cannot be associated with a lower energy balance and/or a decreased muscle glycogen level compared to the CON condition.

Running transiently increases the serum iron level, and this is attributable to hemolysis [53]. Furthermore, an elevated iron level, per se, upregulates hepcidin production [54]. Thus, the post-exercise hepcidin elevation may reflect the iron homeostasis. Exercise-induced IL-6 elevation is the most significant factor for increasing the post-exercise hepcidin level. However, Peeling et al. [28] recently reported that iron status plays a more important role in the post-exercise hepcidin elevation seen in elite athletes; lower serum ferritin and iron levels attenuate the exercise-induced rise in hepcidin. Therefore, an elevated iron level due to iron supplementation in the Fe condition may explain the observed increase in the hepcidin level. However, the serum iron levels were elevated in both conditions during days 2–4. Moreover, the serum ferritin levels did not differ significantly between the conditions, suggesting that the altered iron status caused by iron supplementation did not affect the hepcidin level in the Fe condition.

We performed a unique exploration of the impact of iron supplementation during training on hepcidin level. During the three consecutive days of training, subjects in the Fe condition received a moderate dose (24 mg) of iron supplementation. The daily absorption of iron supplements ranges from 2.3% to 8.5% when the supplement is taken with and without food, respectively [55]. Thus, only small proportions of the iron are absorbed. Higher iron doses may be toxic, as iron catalyzes the production of reactive oxygen products. Hence, iron supplementation theoretically increases the hepcidin level [56]. An earlier study found that higher-dose iron (>40 mg) augmented the hepcidin level in females [23]. Moreover, Deli et al. [57] indicated that a moderate dose of iron supplementation over three weeks promoted pro-oxidant action, and augmented inflammation. Although the detailed mechanism still remains unclear, the findings in the Fe condition on day 4 suggests that even a moderate dose of iron supplementation during short-term endurance training may augment the hepcidin level.

One limitation of our present study is the relatively small sample size. However, we confirmed that the hepcidin level was significantly elevated in the Fe condition; the ES for reflecting condition difference was sufficient (*p* = 0.025, ES = 0.90). Another limitation is the short-term (three consecutive days) nature of the training; further work is needed to explore whether our findings are relevant to long-term training. We used short-term endurance training in an effort to mimic a realistic situation featuring a rapid increase in training stress. Moreover, since we strictly controlled daily energy and iron intake, it was not easy to prepare a long–term training period. Additionally, long-term iron supplementation may be associated with a risk of gastrointestinal disease. 

## 5. Conclusions

The hepcidin level was significantly elevated after three consecutive days of endurance training in subjects taking moderate (24 mg/day) iron supplementation. The results suggest that moderate doses of iron supplementation during consecutive days of endurance training may increase resting hepcidin levels. The present findings would also provide an important message that even a moderate dose of iron supplementation during endurance training may not be a recommendable treatment to improve physical condition in athletes. Future work should explore effect of long-term iron supplementation during training on hepcidin. Moreover, whether other nutritional interventions (e.g., increased CHO intake, antioxidant supplementation) during training attenuate the rise of hepcidin level in endurance athletes would be a valuable topic to explore.

## Figures and Tables

**Table 1:** Running distances and HR during training period.

	Condition	Day 1			Day 2			Day 3		
Running distance	Fe	34.5	±	0.9	34.5	±	0.9	34.5	±	0.9
(km)	CON	33.3	±	0.7	33.3	±	0.7	33.3	±	0.7
Heart rate (bpm)										
Ex1	Fe	158	±	4	157	±	4	154	±	3
	CON	156	±	5	156	±	4	154	±	2
Ex2	Fe	158	±	4	154	±	4	156	±	4
	CON	159	±	5	158	±	3	153	±	3

The values are means ± SE.

**Table 2:** Resting serum ferritin, iron, TSAT during training period (days 1–3) and on the following day after training period.

	Condition	Day 1			Day 2			Day 3			Day 4		
Ferritin	Fe	47.9	±	9.3	52.2	±	9.3	56.9	±	9.0	61.4	±	10.0
(ng/mL)	CON	38.0	±	9.5	39.8	±	10.8	44.9	±	11.2	47.6	±	11.1
Iron	Fe	89.1	±	11.5	115.6	±	10.0	120.1	±	9.3	91.3	±	13.5
(µg/dL)	CON	73.3	±	15.8	143.1	±	23.3	120.1	±	13.9	129.7	±	7.5
TSAT	Fe	31.4	±	5.0	40.9	±	3.5	44.9	±	5.6	34.1	±	6.0
(%)	CON	24.4	±	4.7	50.2	±	10.1	42.4	±	6.2	46.5	±	3.7

The values are means ± SE. Serum ferritin: interaction (condition × time): *p* = 0.504 (ES = 0.005), main effect for condition: *p* = 0.445 (ES = 0.05), main effect for time: *p* = 0.008 (ES = 0.47). Serum iron: interaction (condition × time): *p* = 0.069 (ES = 0.19), main effect for condition (*p* = 0.321, ES = 0.08), main effect for time: *p* = 0.001 (ES = 0.36). Serum TSAT: interaction: *p* = 0.056 (ES = 0.19), main effect for condition: *p* = 0.598 (ES = 0.02), main effect for time: *p* = 0.001 (ES = 0.38).

**Table 3:** Scores of fatigue and muscle soreness during training period.

	Condition	Day 1			Day 2			Day 3			Day 4		
Fatigue	Fe	25	±	7	30	±	7	41	±	10	42	±	6
(mm)	CON	26	±	4	39	±	9	43	±	7	42	±	7
Muscle Soreness	Fe	16	±	5	36	±	6	57	±	9	55	±	7
(mm)	CON	23	±	4	43	±	9	59	±	5	53	±	7

The values are means ± SE. Fatigue: interaction (condition × time): *p* = 0.889 (ES = 0.02), main effect for condition: *p* = 0.690 (ES = 0.01), main effect for time: *p* = 0.020 (ES = 0.61). Muscle soreness; interaction (condition × time): *p* = 0.881 (ES = 0.02), main effect for condition: *p* = 0.498 (ES = 0.04), main effect for time: *p* < 0.001 (ES = 0.55).

**Table 4:** Total energy and macronutrient intakes during training period.

	Condition	Day 1			Day 2			Day 3		
Total energy	Fe	10,850	±	130	11,005	±	110	10,761	±	136
(KJ)	CON	10,560	±	65	10,621	±	74	10,661	±	71
CHO	Fe	395.3	±	7.8	402.5	±	7.7	388.2	±	6.8
(g)	CON	378.4	±	3.4	381.0	±	3.7	383.7	±	3.7
Protein	Fe	87.0	±	0.2	87.9	±	0.8	88.9	±	1.4
(g)	CON	86.8	±	0.2	88.0	±	0.7	87.2	±	0.2
Fat	Fe	65.2	±	0.04	65.9	±	0.47	65.3	±	0.04
(g)	CON	65.2	±	0.04	65.2	±	0.04	65.3	±	0.04
Iron	Fe	30.5	±	0.01	30.5	±	0.01	30.5	±	0.01
(mg)	CON	6.5	±	0.01	6.5	±	0.01	6.5	±	0.01

The values are means ± SE.

## Abbreviations

IL-6	interleukin-6
Hb	hemoglobin
TIBC	total iron-binding capacity
CK	creatine kinase
TSAT	transferrin saturation
CV	coefficients of variation
ES	effect size
